# Supplementary material for: Furosemide and the Symptom Burden: The Potential Mediating Role of Uremic Toxins in Patients with CKD
Source: Toxins (Basel). 2025 Nov 1;17(11):541. doi: 10.3390/toxins17110541 (PMC12656462; doi:10.3390/toxins17110541)
Supplement: Supplementary file 1 [file toxins-17-00541-s001.zip › toxins-3871452-supplementary.pdf]

Supplementary Materials: Furosemide and the Symptom Burden: The Potential Mediating Role of Uremic Toxins in Patients with CKD

Margaux Costes-Albrespic, Sophie Liabeuf, Islam-Amine Larabi, Solène M. Laville, Bénédicte Stengel, Abdou Y. Omorou, Luc Frimat, Jean-Claude Alvarez, Ziad A. Massy, Natalia Alencar de Pinho and the CKD-REIN Study Group

First, we assessed the association between furosemide dose category and "symptom score 8". We hypothesized that the higher the dose of furosemide in CKD patients, the lower the symptom score (i.e. the higher the symptom burden), independently of confounding factors such as kidney function and comorbidities. We also hypothesized that this association would be blunted by additional adjustment for the sum of UTs competing for excretion pathways in the proximal tubule (i.e. IS, KYN, PCS, and IAA), reflecting a potential mediation effect (Baron & Kenny method).24 Beta regression models with a logit link were used to assess this association at baseline, given symptom score's left-skewed distribution. Since beta regression requires values strictly between 0 and 1 (excluding the boundaries), the "symptom score 8" (ranging from 0 to 100) was divided by 100 (Y′) and then transformed using the formula: $Y'' = Y'(n - 1 + 0.5) / n$, where n is the sample size.25 We first adjusted the model for clinically relevant factors identified from the literature. These included age (natural splines, knots at 50, 70 and 80 years), sex, obesity, log-uACR, eGFR, smoking status, haemoglobin concentration, diabetes, history of HF, CAD, cerebrovascular disease, PAD, and the number of co-prescribed medications. We then ran a second model with further adjustment for log- ΣUTs to obtain a preliminary assessment of mediation. We examined whether this association differed according to sex including a multiplicative interaction term between sex and furosemide dose. Ordinal logistic regressions were used to assess the association between furosemide dose and each item of the "symptom score 11". Each item was treated as an ordinal variable reflecting symptom severity, with the categories "very much" and "extremely" combined into a single category. The proportional odds assumption was tested using likelihood ratio test and sex was retained as a nominal effect. Global p-values were adjusted for multiple comparisons using the Holm-Bonferroni26 method.

Finally, mediation analyses were conducted to assess total, direct and indirect effects of furosemide on "symptom score 8" mediated through IS, KYN, PCS, IAA or the ΣUTs. We hypothesized that patients prescribed furosemide would have increased UT levels, and, consequently, a higher "symptom score 8". Structural equation modelling (SEM) was used and implemented with the lavaan package in R27. We applied the maximum likelihood (ML) estimation method with nonparametric bootstrapping (1,000 samples), which makes no assumptions about the underlying distribution of the data.28,29 Direct, total and indirect effects were significant if their 95% CI did not include zero. In the setting of structural equation modelling, it is not required to pre-test the X→M and M→Y paths for statistical significance before conducting the mediation analysis, unlike the classical Baron & Kenny approach. Modern approaches (e.g., Preacher & Hayes, structural equation modelling with bootstrap) recommend testing the indirect effect without this intermediary analysis, as the mediation effect can be present even if individual paths are not statistically significant. Given that furosemide doses >120 mg were independently associated with higher serum UT concentrations, and that SEM supports only binary and continuous independent variables without smooth functions, we categorized furosemide doses into two groups: ≤120 mg and >120 mg.27 Two regression models were specified within the SEM framework: firstly, a model for "symptom score 8" adjusted for the concerned log-UT and covariates used in the beta regression (smooth function for age excluded); secondly, a model for the concerned log-UT concentration, adjusted for the same covariates (haemoglobin excluded), log- CRP, and serum albumin.

As sensitivity analyses, we repeated the beta regression model using the furosemide dose as a continuous variable with a smooth function (natural splines, with knots at 40, 80, 100 and 120 mg). We also included an interaction term between furosemide dose category and sex, given that the symptom score differs between sexes.

SEM were also repeated with the continuous (for an increase of 20 mg) and the ordinal (for an increase of one category, out of four) forms of furosemide dose.

Assuming data missing at random, we performed multiple imputation with chained equations (MICE), implemented with the MICE package in R software (version 4.1.2).30,31 Beta regression and ordinal logistic regression models were generate for each of the 30 generated datasets; pooled regression coefficients were obtained according to Rubin's rules. In mediation models, Full Information Maximum Likelihood method was used instead. The threshold for statistical significance was set to p<0.05. All statistical analyses were performed with R software (version 4.1.2).

Table S1. Patient's characteristics at baseline with information on missing data, overall and by furosemide dose category.

		Furosemide prescription			
**Characteristic**	**Overall*** **N = 2,053**	**None*** **N = 1,357**	**10-40 mg/day*** **N = 400**	**60-120 mg/day*** N = 165	**>120 mg/day*** N = 131
Age (years), median [IQR]	68 [61; 76] (0, 0)	67 [58; 74] (0, 0)	71 [65; 79] (0, 0)	72 [66; 80] (0, 0)	71 [67; 77] (0, 0)
Men	1,358 (66%), (0, 0)	886 (65%), (0, 0)	264 (66%), (0, 0)	114 (69%), (0, 0)	94 (72%), (0, 0)
Smoker	232 (11%), (11, 1)	164 (12%), (6, 0)	39 (10%), (3, 1)	17 (10%), (1, 1)	12 (9%), (1, 1)
Education (years)					
< 9	276 (14%), (20, 1)	142 (11%), (13, 1)	66 (17%), (5, 1)	41 (25%), (1, 1)	27 (21%), (1, 1)
9-11	1,019 (50%), (20, 1)	636 (47%), (13, 1)	214 (54%), (5, 1)	92 (56%), (1, 1)	77 (59%), (1, 1)
≥ 12	738 (36%), (20, 1)	566 (42%), (13, 1)	115 (29%), (5, 1)	31 (19%), (1, 1)	26 (20%), (1, 1)
eGFR (mL/min/1.73m2), mean (SD)	35 (13) (11, 0.5)	37 (13) (7, 0.5)	31 (12) (2, 0.5)	29 (11) (1, 0.6)	28 (11) (1, 0.8)
Albumin-to-creatinine ratio (mg/g), median [IQR]	104 [20; 496] (305, 15)	81 [17; 455] (202, 15)	133 [24; 531] (63, 16)	207 [34; 768] (24, 15)	230 [42; 778] (16, 12)
Diabetes	818 (40%), (5, 0)	423 (31%), (2, 0)	197 (49%), (2, 1)	102 (62%), (1, 1)	96 (73%), (0, 0)
Cardiovascular	1,067 (52%), (12, 1)	559 (41%), (8, 1)	272 (68%), (2, 1)	124 (76%), (2, 1)	112 (85%), (0, 0)
Coronary artery disease	489 (24%), (16, 1)	225 (17%), (9, 1)	129 (33%), (5, 1)	73 (45%), (1, 1)	62 (48%), (1, 1)
Heart failure	261 (13%), (3, 0)	73 (5%), (3, 0)	76 (19%), (0, 0)	46 (28%), (0, 0)	66 (50%), (0, 0)
Cerebrovascular disease	220 (11%), (17, 1)	122 (9%), (11, 1)	47 (12%), (3, 1)	28 (17%), (2, 1)	23 (18%), (1, 1)
Peripheral artery disease	320 (16%), (15, 1)	151 (11%), (11, 1)	91 (23%), (3, 1)	43 (26%), (0, 0)	35 (27%), (1, 1)
BMI (kg/m2), mean (SD)	28.7 (5.8) (35, 1.7)	27.6 (5.2) (26, 1.9)	29.9 (6.2) (7, 1.8)	31.6 (6.6) (2, 1.2)	33.2 (6.1) (0, 0)
Obesity (BMI ≥ 30 kg/m2)	711 (35%), (35, 2)	362 (27%), (26, 2)	175 (45%), (7, 2)	85 (52%), (2, 1)	89 (68%), (0, 0)
C-reactive protein (mg/L), median [IQR]	2.3 [1.1; 5.0] (105, 5.1)	2.0 [0.9; 4.2] (49, 3.6)	2.6 [1.3; 5.4] (24, 6.0)	2.8 [1.5; 6.7] (18, 11)	5.1 [2.0; 9.1] (14, 11)

	Furosemide prescription				
**Characteristic**	**Overall*** **N = 2,053**	**None*** **N = 1,357**	**10-40 mg/day*** **N = 400**	**60-120 mg/day*** N = 165	**>120 mg/day*** N = 131
Serum albumin (g/L), median [IQR]	41.0 [38.5; 43.3] (7, 0.3)	41.3 [39.0; 43.6] (7, 0.5)	40.6 [37.9; 42.7] (0, 0)	40.0 [37.5; 42.5] (0, 0)	39.0 [35.6; 41.5] (0, 0)
Kalemia (mmol/L), mean (SD)	4.53 (0.51) (6, 0.3)	4.54 (0.50) (5, 0.4)	4.61 (0.54) (1, 0.3)	4.45 (0.51) (0, 0)	4.29 (0.51) (0, 0)
Calcemia (mmmol/L), mean (SD)	2.35 (0.13) (66, 3.2)	2.36 (0.12) (45, 3.3)	2.35 (0.14) (8, 2.0)	2.34 (0.14) (9, 5.5)	2.29 (0.18) (4, 3.1)
Hemoglobine (g/dL), mean (SD)	13.09 (1.66) (30, 1.5)	13.29 (1.62) (24, 1.8)	12.78 (1.79) (4, 1.0)	12.90 (1.47) (2, 1.2)	12.35 (1.57) (0, 0)
Systolic blood pressure (mm Hg), mean (SD)	141 (20) (22, 1.1)	140 (19) (15, 1.1)	144 (21) (5, 1.3)	143 (23) (0, 0)	143 (21) (2, 1.5)
Diastolic blood pressure (mm Hg), mean (SD)	78 (12) (23, 1.1)	79 (12) (16, 1.2)	77 (11) (5, 1.3)	75 (13) (0, 0)	74 (12) (2, 1.5)
Number of drugs prescribed, median [IQR]	8 [5; 10] (8, 0.4)	6 [4; 9] (8, 0.6)	9 [8; 12] (0, 0)	10 [8; 13] (0, 0)	12 [9; 14] (0, 0)
Depression score (CES-D), median [IQR]	7.0 [4.0; 10.0] (35, 1.7)	6.0 [3.3; 10.0] (20, 1.5)	7.0 [4.0; 10.0] (7, 1.8)	8.0 [4.0; 11.0] (7, 4.2)	9.0 [6.0; 12.0] (1, 0.8)
Adherence					
good	820 (40%), (11, 1)	567 (42%), (7, 1)	154 (39%), (2, 1)	62 (38%), (1, 1)	37 (28%), (1, 1)
moderate	1,090 (53%), (11, 1)	698 (52%), (7, 1)	220 (55%), (2, 1)	89 (54%), (1, 1)	83 (64%), (1, 1)
poor	132 (6%), (11, 1)	85 (6%), (7, 1)	24 (6%), (2, 1)	13 (8%), (1, 1)	10 (8%), (1, 1)
Ureamic toxins, median [IQR]					
Indoxyl sulphate (µM)	0.24 [0.13; 0.44] (0, 0)	0.21 [0.12; 0.38] (0, 0)	0.28 [0.16; 0.50] (0, 0)	0.33 [0.17; 0.63] (0, 0)	0.45 [0.23; 0.80] (0, 0)
P-cresyl sulphate (µM)	0.99 [0.45; 1.90] (0, 0)	0.82 [0.37; 1.57] (0, 0)	1.33 [0.65; 2.19] (0, 0)	1.36 [0.86; 2.93] (0, 0)	1.65 [0.80; 3.46] (0, 0)
Kynurenine (µM)	0.61 [0.43; 0.86] (0, 0)	0.56 [0.40; 0.79] (0, 0)	0.71 [0.51; 0.94] (0, 0)	0.75 [0.55; 0.96] (0, 0)	0.85 [0.59; 1.22] (0, 0)
Indole-3-Acetic Acid (µM)	0.18 [0.13; 0.29] (0, 0)	0.18 [0.13; 0.27] (0, 0)	0.18 [0.14; 0.29] (0, 0)	0.22 [0.14; 0.33] (0, 0)	0.26 [0.17; 0.35] (0, 0)
∑PBUTs (µM)	2.15 [1.37; 3.51] (0, 0)	1.85 [1.22; 3.02] (0, 0)	2.57 [1.68; 4.23] (0, 0)	2.87 [1.97; 4.67] (0, 0)	3.44 [2.28; 5.30] (0, 0)
Symptom score 11, median [IQR]	76 (16) (0, 0)	77 (16) (0, 0)	74 (16) (0, 0)	72 (16) (0, 0)	67 (18) (0, 0)
Symptom score 8, median [IQR]	76 (17) (0, 0)	78 (16) (0, 0)	76 (17) (0, 0)	73 (17) (0, 0)	68 (18) (0, 0)

Table S2. Association between symptom score and furosemide dose category, stratified by sex.

	Exp(estimate) (IC 95%)		P value of the interaction
	Female	Male	
Crude			0.541
10-40 mg	**0.811 (0.690-0.953)**	0.935 (0.763-1.147)	
60-120 mg	**0.740 (0.581-0.943)**	0.797 (0.592-1.073)	
>120 mg	**0.567 (0.431-0.747)**	**0.547 (0.393-0.760)**	
Adjusted*			0.398
10-40 mg	0.948 (0.806-1.115)	1.104 (0.903-1.348)	
60-120 mg	0.861 (0.675-1.099)	0.989 (0.737-1.326)	
>120 mg	0.755 (0.570-1.000)	0.726 (0.525-1.003)	
Adjusted* + ∑UTs			0.373
10-40 mg	0.948 (0.806-1.115)	1.111 (0.910-1.358)	
60-120 mg	0.865 (0.678-1.103)	0.996 (0.743-1.336)	
>120 mg	0.757 (0.572-1.002)	0.736 (0.532-1.017)	

For each model, sex estimates were calculated based on the interaction term between furosemide dose and sex. Exponentiated estimates from beta regression with a logit link are interpretable as the ratio (mean symptom score)/(1- mean symptom score). Values <1 indicate a lower symptom score (i.e., higher symptom burden), while values >1 indicate a higher symptom score (i.e., lower symptom burden). ∑UTs were log transformed.

*Adjusted for age (natural splines, knots at 50, 70 and 80 years), sex, obesity, log-uACR, eGFR, smoking status, haemoglobin concentration, diabetes, history of heart failure, coronary heart disease, cerebrovascular disease, peripheral artery disease, and the number of prescribed medications.

Abbreviations: CI, confidence interval; eGFR, estimated glomerular filtration rate; uACR, urinary albumin-to-creatinine ratio; ∑UTs, sum of the free concentrations of indoxyl sulphate, kynurenine, p-cresyl sulphate and indole-3-acetic acid.

Table S3. Association between symptom score and furosemide dose category, further adjusted.

	Exp(estimate) (IC 95%), Reference: no furosemide prescription
**Adjusted***	
**10-40 mg**	1.04 (0.94-1.15)
**60-120 mg**	0.94 (0.81-1.09)
**>120 mg**	0.73 (0.62-0.87)
**Adjusted* - haemoglobin concentration**	
**10-40 mg**	1.03 (0.93-1.15)
**60-120 mg**	0.94 (0.81-1.09)
**>120 mg**	0.72 (0.61-0.85)
**Adjusted* + education + adherence**	
**10-40 mg**	1.03 (0.93-1.14)
**60-120 mg**	0.94 (0.81-1.09)
**>120 mg**	0.74 (0.62-0.87)
**Adjusted* + systolic BP**	
**10-40 mg**	1.04 (0.94-1.15)
**60-120 mg**	0.94 (0.81-1.09)
**>120 mg**	0.73 (0.62-0.86)
**Adjusted* + serum calcium + serum potassium**	
**10-40 mg**	1.04 (0.94-1.15)
**60-120 mg**	0.95 (0.82-1.11)
**>120 mg**	0.73 (0.62-0.87)

Exponentiated estimates from beta regression with a logit link are interpretable as the ratio (mean symptom score)/(1- mean symptom score). Values < 1 indicate a lower symptom score (i.e., higher symptom burden), while values > 1 indicate a higher symptom score (i.e., lower symptom burden).

*Adjusted for age (natural splines, knots at 50, 70 and 80 years), sex, obesity, log-uACR, eGFR, smoking status, diabetes, history of heart failure, coronary heart disease, cerebrovascular disease, peripheral disease, number of co-prescribed medications and haemoglobin concentration.

Education was categorized in three groups: <9, 9-11, ≥12 years of schooling. Adherence was assessed using the Girerd Score and categorized in 3 groups: good, moderate and poor.

Abbreviations: CI, confidence interval; eGFR, estimated glomerular filtration rate; uACR, urinary albumin-to-creatinine ratio.

Table S4. Adjusted odds ratio of being more bothered by a symptom, associated with furosemide dose category. Reference: no furosemide prescription.

	Odds ratio (95% CI)			
**Furosemide doses**	**10-40 mg**	**60-120 mg**	>120 mg	P value<br>P value adjusted*
Soreness in muscles	0.92 [0.74-1.15]	1.09 [0.79-1.51]	0.96 [0.66-1.40]	0.787<br>1.000
Cramps	1.03 [0.83-1.28]	0.97 [0.71-1.33]	0.96 [0.66-1.39]	0.975<br>1.000
Itchy skin	0.81 [0.64-1.02]	1.05 [0.76-1.46]	1.40 [0.96-2.06]	**0.035**<br>0.240
Dry Skin	0.97 [0.77-1.21]	1.19 [0.87-1.64]	**1.89 [1.30-2.73]**	**0.005**<br>**0.045**
Faintness or dizziness	0.93 [0.73-1.18]	1.31 [0.93-1.83]	1.27 [0.85-1.88]	0.189<br>0.880
Lack of appetite	0.89 [0.68-1.16]	0.91 [0.62-1.33]	**1.65 [1.09-2.49]**	**0.030**<br>0.240
Numbness in hands or feet	1.13 [0.90-1.41]	1.02 [0.74-1.42]	**2.19 [1.51-3.17]**	**0.000**<br>**0.000**
Nausea or upset stomach	**0.69 [0.52-0.91]**	0.97 [0.66-1.42]	1.12 [0.72-1.72]	**0.034**<br>0.240
Chest pain	1.17 [0.88-1.55]	1.30 [0.87-1.93]	**1.60 [1.03-2.51]**	0.176<br>0.880
Shortness of breath	**1.41 [1.13-1.76]**	1.18 [0.86-1.63]	**1.86 [1.28-2.71]**	**0.001**<br>**0.010**
Washed out or drained	1.00 [0.81-1.25]	1.11 [0.80-1.54]	1.31 [0.90-1.91]	0.510<br>1.000

Odds ratios of being more bothered by a symptom estimated with ordinal logistic regression for patients prescribed a given dose category of furosemide, relative to those without furosemide prescription. Models ware adjusted for the ∑UTs, age (natural splines, knots at 50, 70 and 80 years), sex, obesity, log-uACR, eGFR, smoking status, haemoglobin concentration, diabetes, history of heart failure, coronary heart disease, cerebrovascular disease, peripheral artery disease, and the number of prescribed medications.

*After Holm-Bonferroni correction for multiple comparisons.

Abbreviations: CI, confidence interval; eGFR, estimated glomerular filtration rate; uACR, urinary albumin-to-creatinine ratio; ∑UTs, sum of the free concentrations of indoxyl sulphate, kynurenine, p-cresyl sulphate and indole-3-acetic acid.

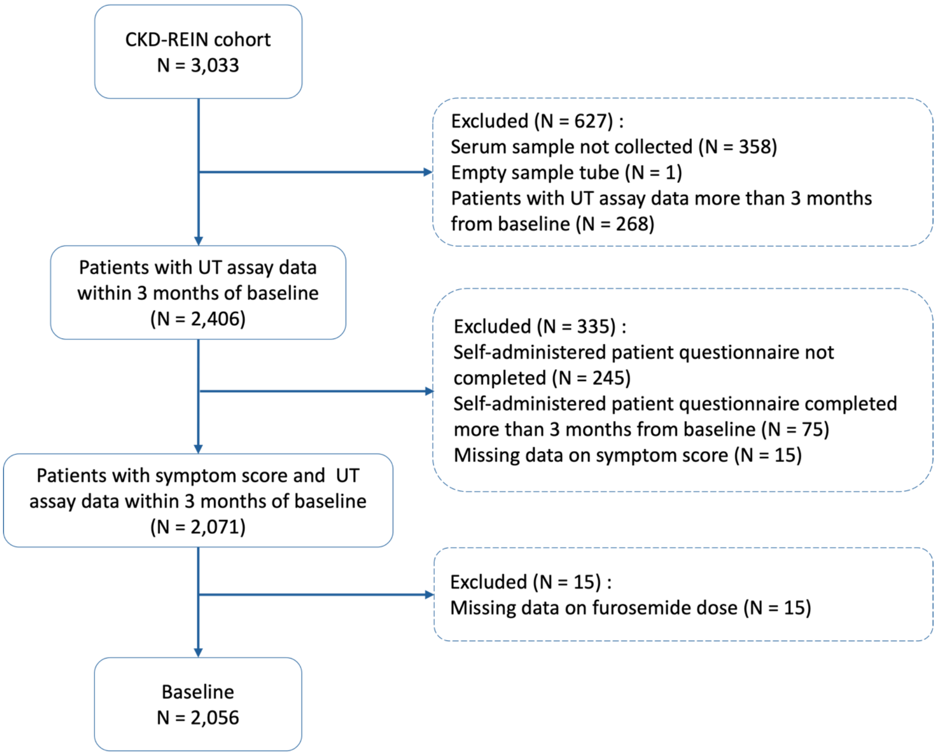

Figure S1. Study flow chart. Abbreviations: UT, uraemic toxins.

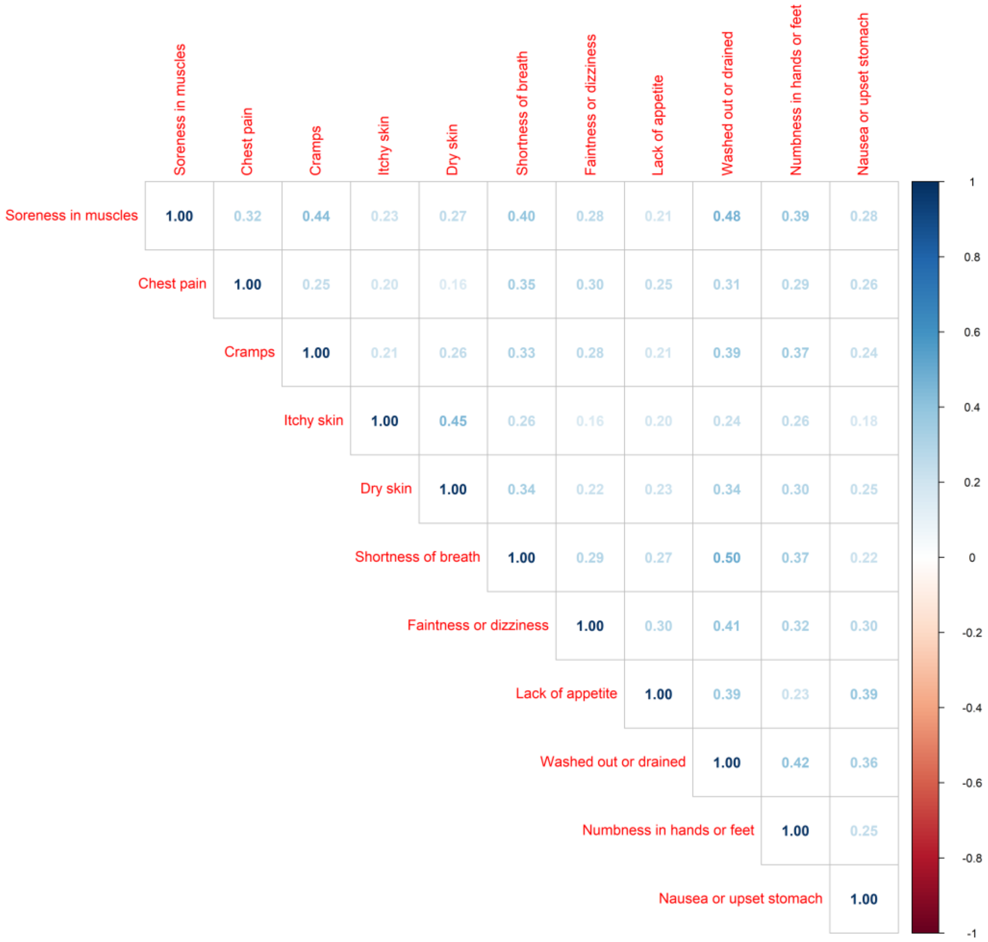

Figure S2. Correlation between items of the symptom score. Spearman's rank correlation method was used.

A.) Adjusted*

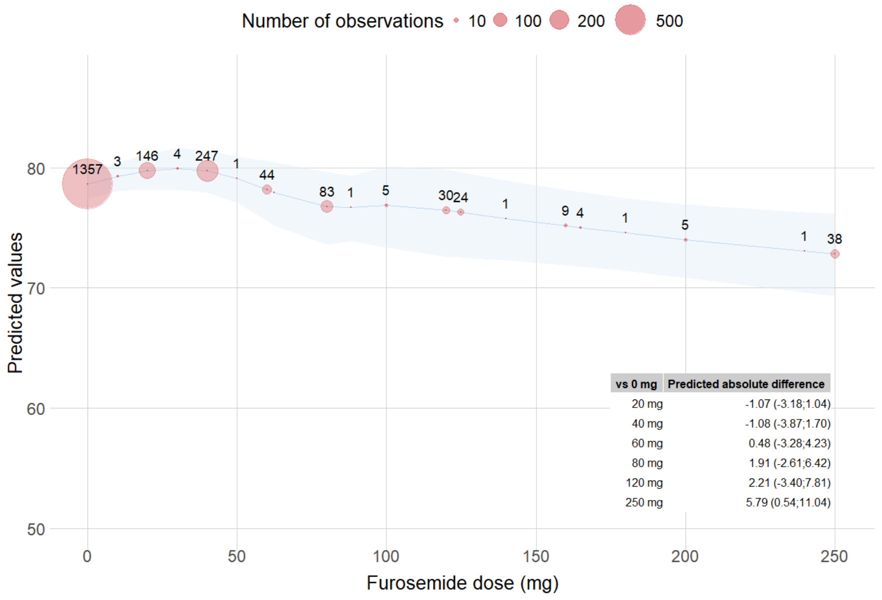

B.) Adjusted* + ∑UTs

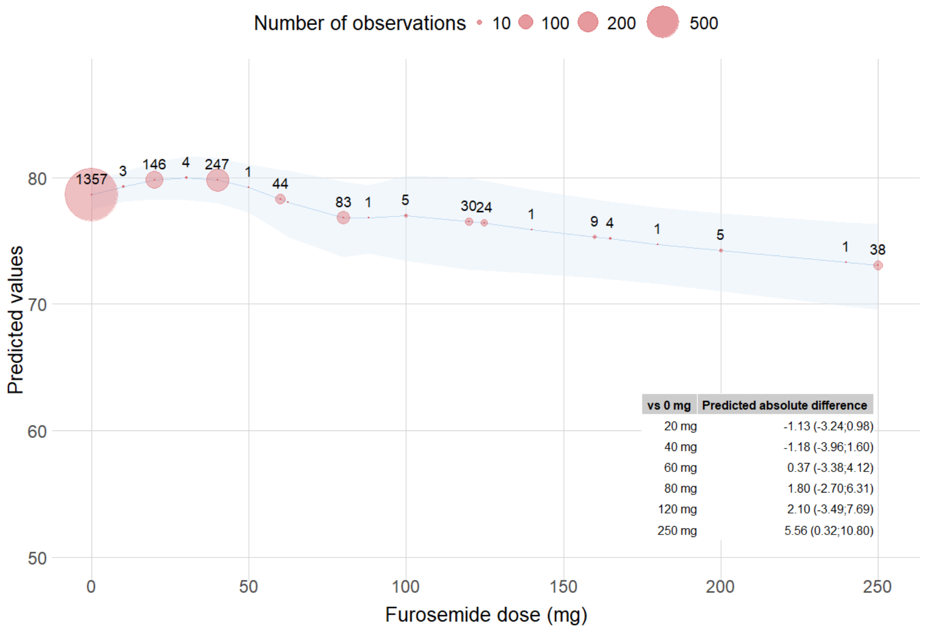

Figure S3. Predicted symptom score as a smooth function of furosemide dose, (A) adjusted and (B) adjusted + ∑UTs. ∑UTs were log transformed. The red circles in the plots are proportional to the number of observations at each data point, with the exact number of observations indicated above each circle. A total of 48 observations are not shown (distributed between 290 mg and 1000 mg). For each plot, a table presents the predicted absolute difference (95% CI) between no furosemide prescription and doses of 20, 40, 60, 80, 120, and 250 mg—doses prescribed to 30 or more patients. Furosemide dose was modelled with natural splines with knots at 40mg, 80mg, 100mg and 120 mg. *The model is adjusted for age (natural splines, knots at 50, 70 and 80 years), sex, obesity, log-uACR, eGFR, smoking status, haemoglobin concentration, diabetes, history of heart failure, coronary heart disease, cerebrovascular disease, peripheral artery disease, and the number of prescribed medications. Abbreviations: CI, confidence interval; eGFR, estimated glomerular filtration rate; uACR, urinary albumin-to-creatinine ratio; ∑UTs, sum of the free concentrations of indoxyl sulphate, kynurenine, p-cresyl sulphate and indole-3-acetic acid.

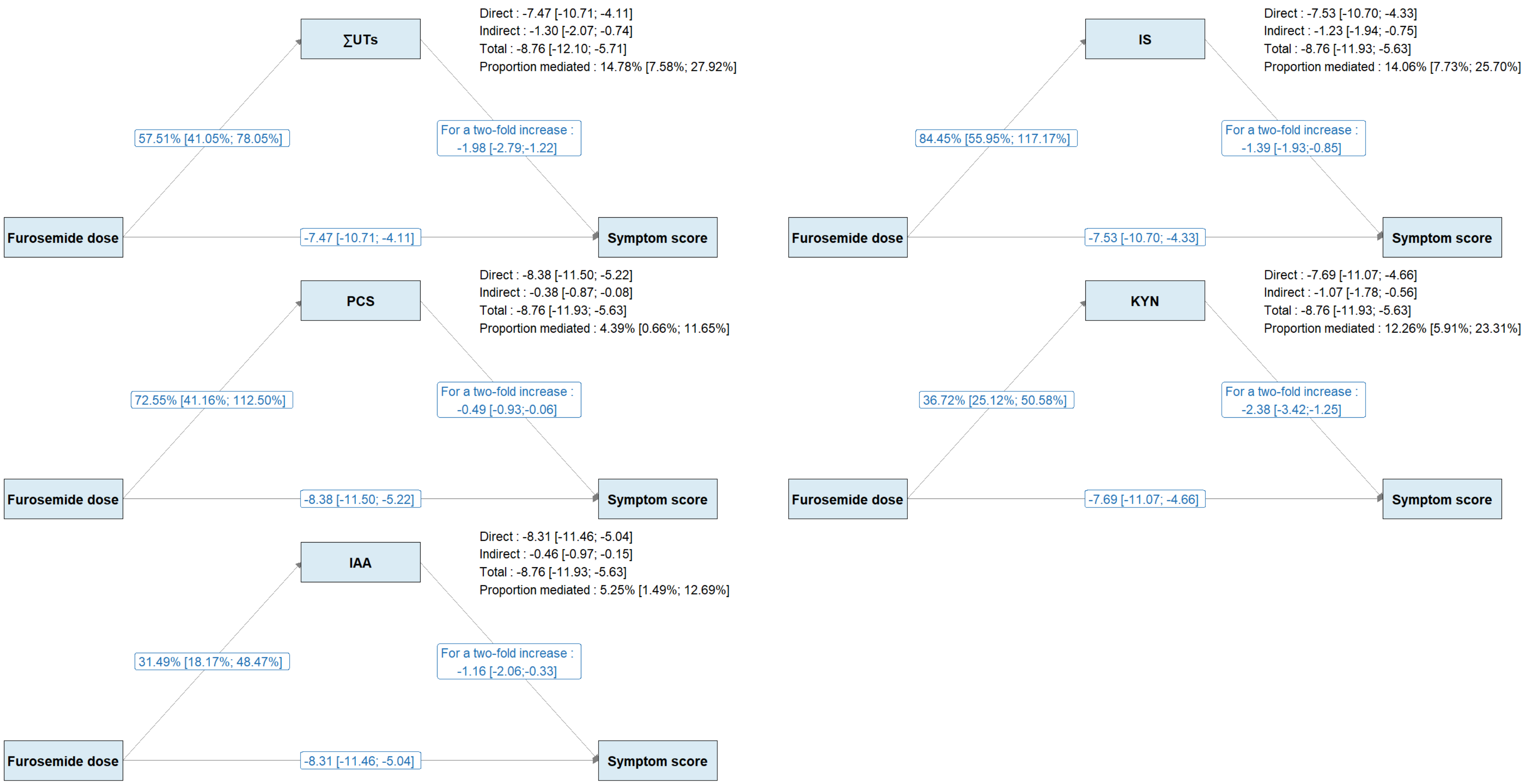

Figure S4. Results of the crude structural equation modelling analysis, with furosemide as a binary variable. Furosemide was treated as a binary variable: ≤120 mg vs. >120 mg. Maximum likelihood estimation with bootstrapping (1000 replications) was used. The symptom score was based on 8 selected symptoms (soreness in muscles, cramps, itchy skin, dry skin, faintness or dizziness, lack of appetite, numbness in hands or feet, and nausea or upset stomach), and UTs were log-transformed. For interpretability, estimates were transformed using either the exponential function or multiplication by log(2), following standard conventions for log-linear models. Models for "symptom score 8" were adjusted for age, sex, obesity, log-uACR, eGFR, smoking status, haemoglobin concentration, diabetes, history of heart failure, coronary heart disease, cerebrovascular disease, peripheral artery disease, and the number of prescribed medications. Models for UT concentration were adjusted for the same covariates (haemoglobin excluded), log- CRP and serum albumin. Abbreviations: eGFR, estimated glomerular filtration rate; IAA, indole-3-acetic acid; IS, indoxyl sulphate; KYN, kynurenine; PCS, p-cresyl sulphate; uACR, urinary albumin-to-creatinine ratio; UTs, uremic toxins; ∑UTs, sum of the free concentrations of IS, KYN, PCS and IAA.

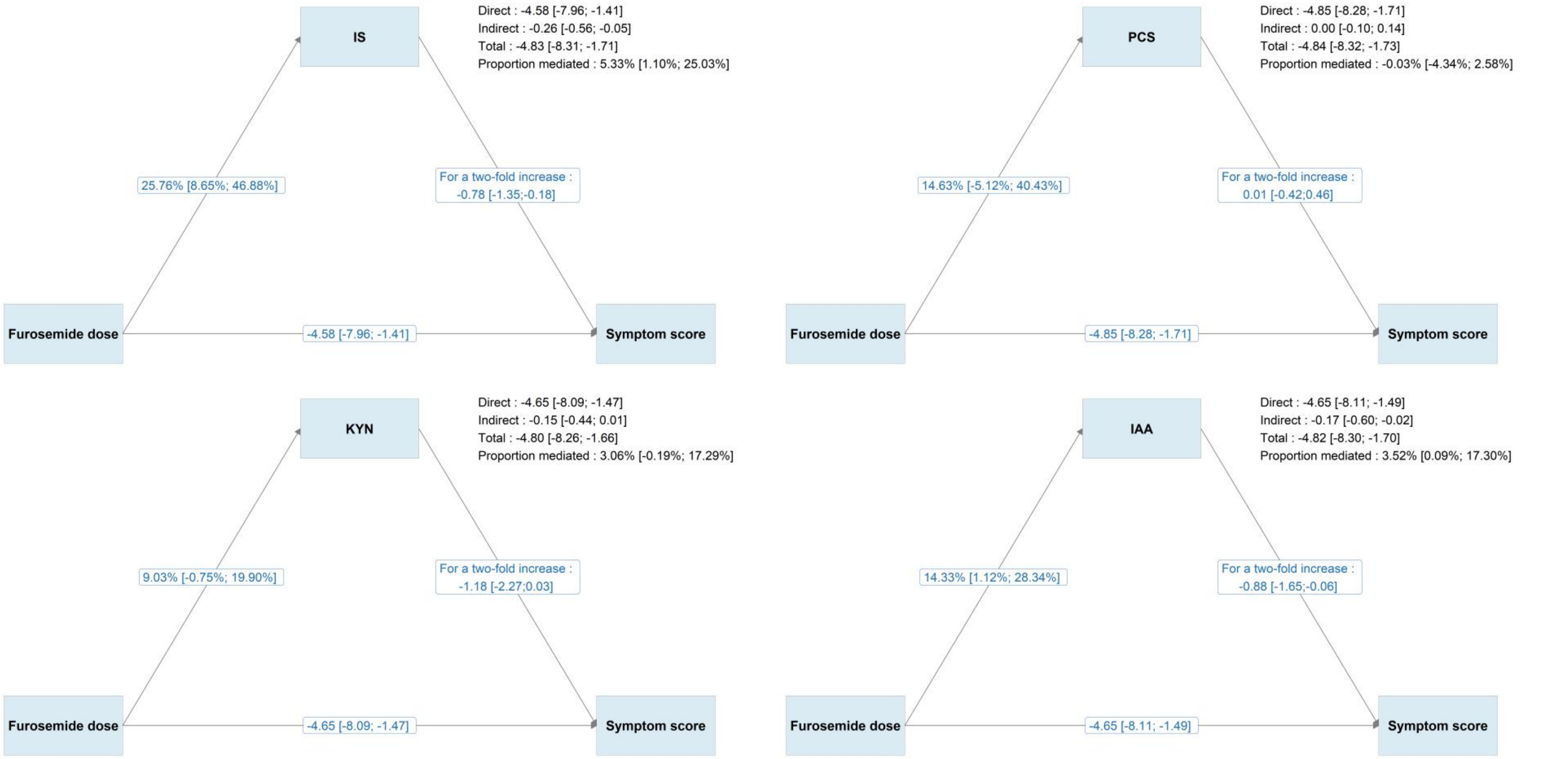

Figure S5.Results of the adjusted structural equation modelling analysis, for each UT and with furosemide as a binary variable. Furosemide was treated as a binary variable: ≤120 mg vs. >120 mg. Maximum likelihood estimation with bootstrapping (1000 replications) was used. The symptom score was based on 8 selected symptoms (soreness in muscles, cramps, itchy skin, dry skin, faintness or dizziness, lack of appetite, numbness in hands or feet, and nausea or upset stomach), and UTs were log-transformed. For interpretability, estimates were transformed using either the exponential function or multiplication by log(2), following standard conventions for log-linear models. Models for "symptom score 8" were adjusted for age, sex, obesity, log-uACR, eGFR, smoking status, haemoglobin concentration, diabetes, history of heart failure, coronary heart disease, cerebrovascular disease, peripheral artery disease, and the number of prescribed medications. Models for UT concentration were adjusted for the same covariates (haemoglobin excluded), log- CRP and serum albumin. Abbreviations: eGFR, estimated glomerular filtration rate; IAA, indole-3-acetic acid; IS, indoxyl sulphate; KYN, kynurenine; PCS, p-cresyl sulphate; uACR, urinary albumin-to-creatinine ratio; UTs, uremic toxins.

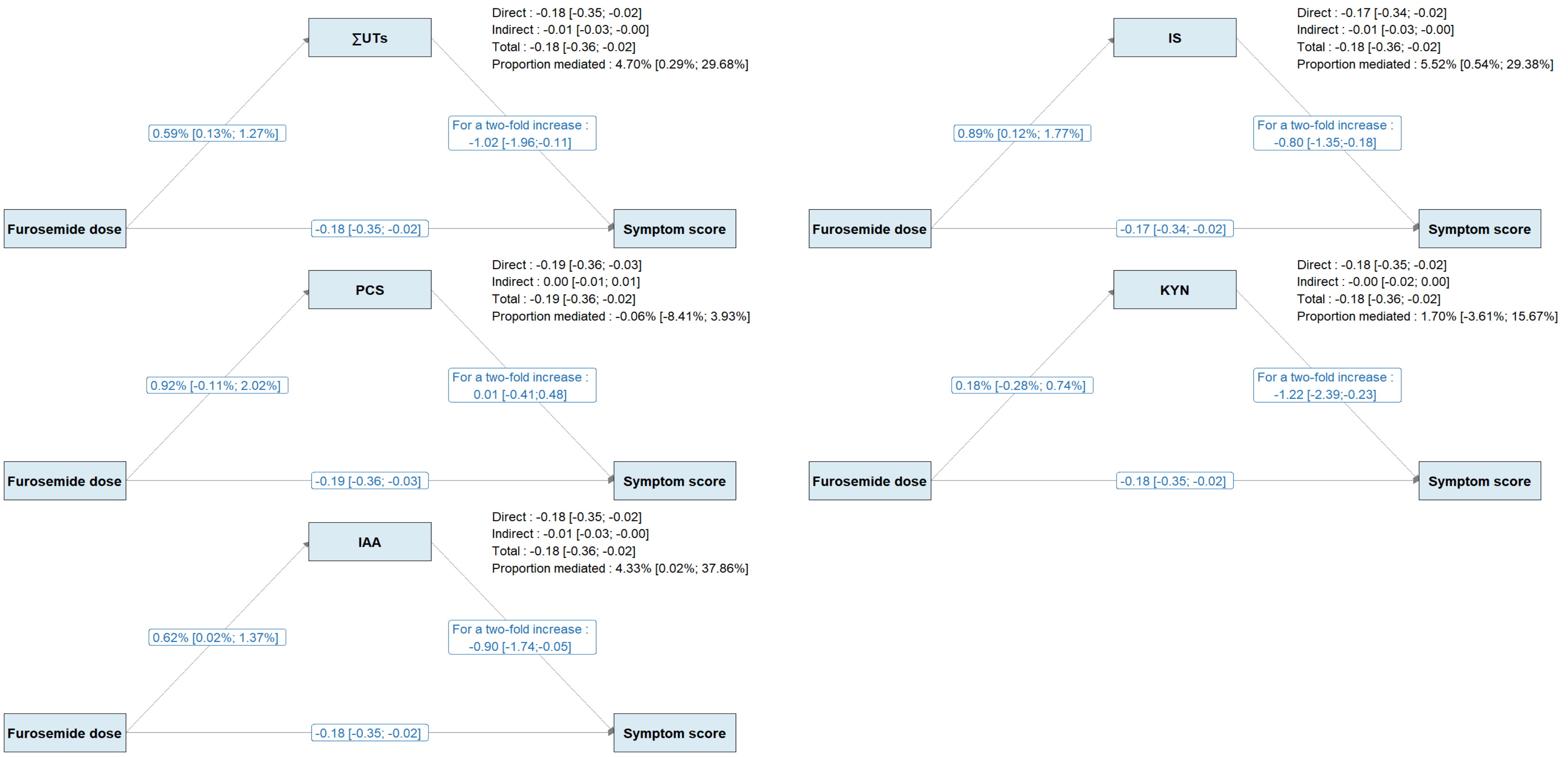

Figure S6. Results of the adjusted structural equation modelling analysis, with furosemide as a continuous variable. Furosemide was treated as continuous, for an increase of 20 mg. Maximum likelihood estimation with bootstrapping (1000 replications) was used. The symptom score was based on 8 selected symptoms (soreness in muscles, cramps, itchy skin, dry skin, faintness or dizziness, lack of appetite, numbness in hands or feet, and nausea or upset stomach), and UTs were log-transformed. For interpretability, estimates were transformed using either the exponential function or multiplication by log(2), following standard conventions for log-linear models. Models for "symptom score 8" were adjusted for age, sex, obesity, log-uACR, eGFR, smoking status, haemoglobin concentration, diabetes, history of heart failure, coronary heart disease, cerebrovascular disease, peripheral artery disease, and the number of prescribed medications. Models for UT concentration were adjusted for the same covariates (haemoglobin excluded), log- CRP and serum albumin. Abbreviations: eGFR, estimated glomerular filtration rate; IAA, indole-3-acetic acid; IS, indoxyl sulphate; KYN, kynurenine; PCS, p-cresyl sulphate; uACR, urinary albumin-to-creatinine ratio; UTs, uremic toxins; ∑UTs, sum of the free concentrations of IS, KYN, PCS and IAA.

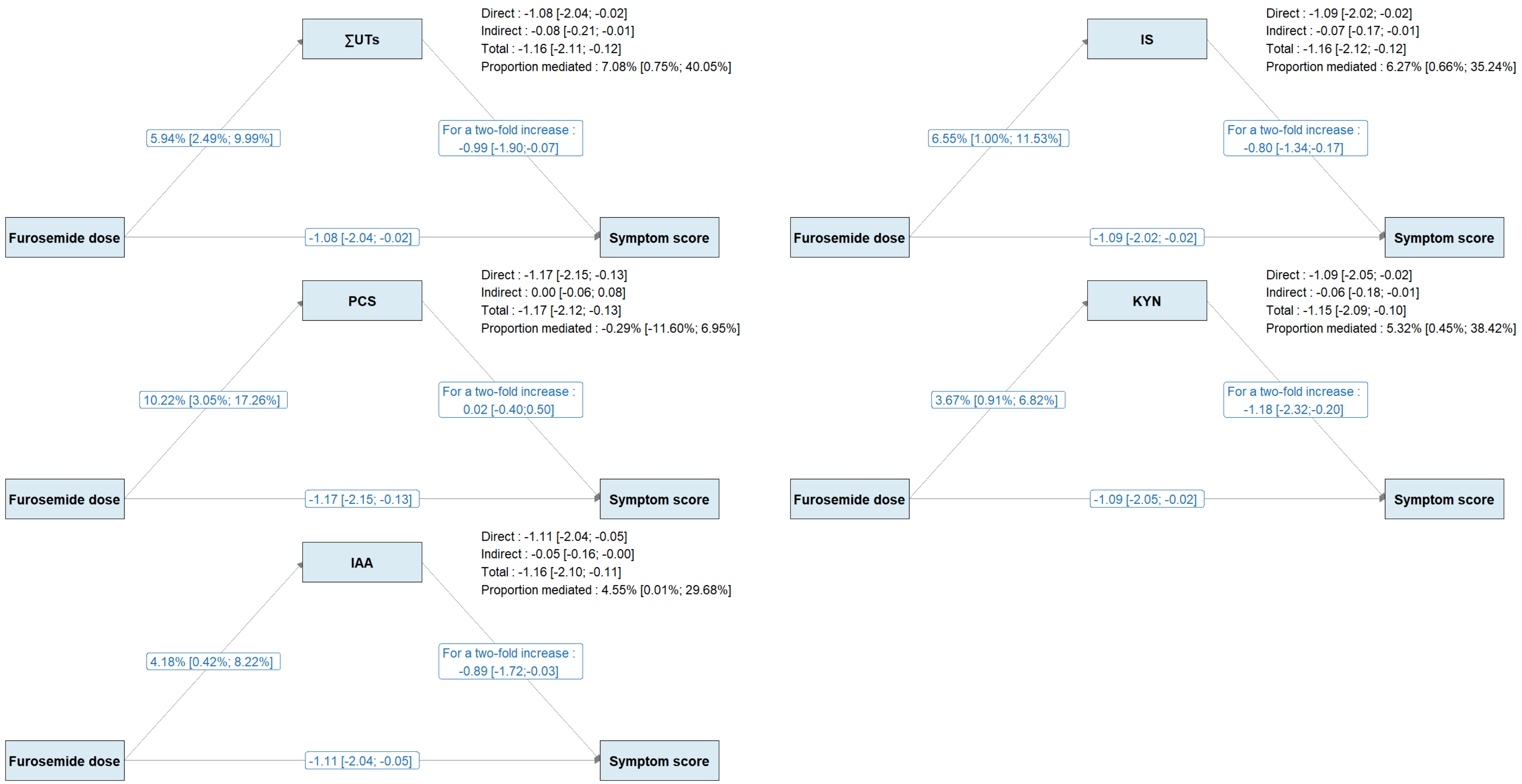

Figure S7. Results of the adjusted structural equation modelling analysis, with furosemide in categories. Furosemide was treated as continuous, for an increase of one category. Maximum likelihood estimation with bootstrapping (1000 replications) was used. The symptom score was based on 8 selected symptoms (soreness in muscles, cramps, itchy skin, dry skin, faintness or dizziness, lack of appetite, numbness in hands or feet, and nausea or upset stomach), and UTs were log-transformed. For interpretability, estimates were transformed using either the exponential function or multiplication by log(2), following standard conventions for log-linear models. Models for "symptom score 8" were adjusted for age, sex, obesity, log-uACR, eGFR, smoking status, haemoglobin concentration, diabetes, history of heart failure, coronary heart disease, cerebrovascular disease, peripheral artery disease, and the number of prescribed medications. Models for UT concentration were adjusted for the same covariates (haemoglobin excluded), log- CRP and serum albumin. Abbreviations: eGFR, estimated glomerular filtration rate; IAA, indole-3-acetic acid; IS, indoxyl sulphate; KYN, kynurenine; PCS, p-cresyl sulphate; uACR, urinary albumin-to-creatinine ratio; UTs, uremic toxins; ∑UTs, sum of the free concentrations of IS, KYN, PCS and IAA.

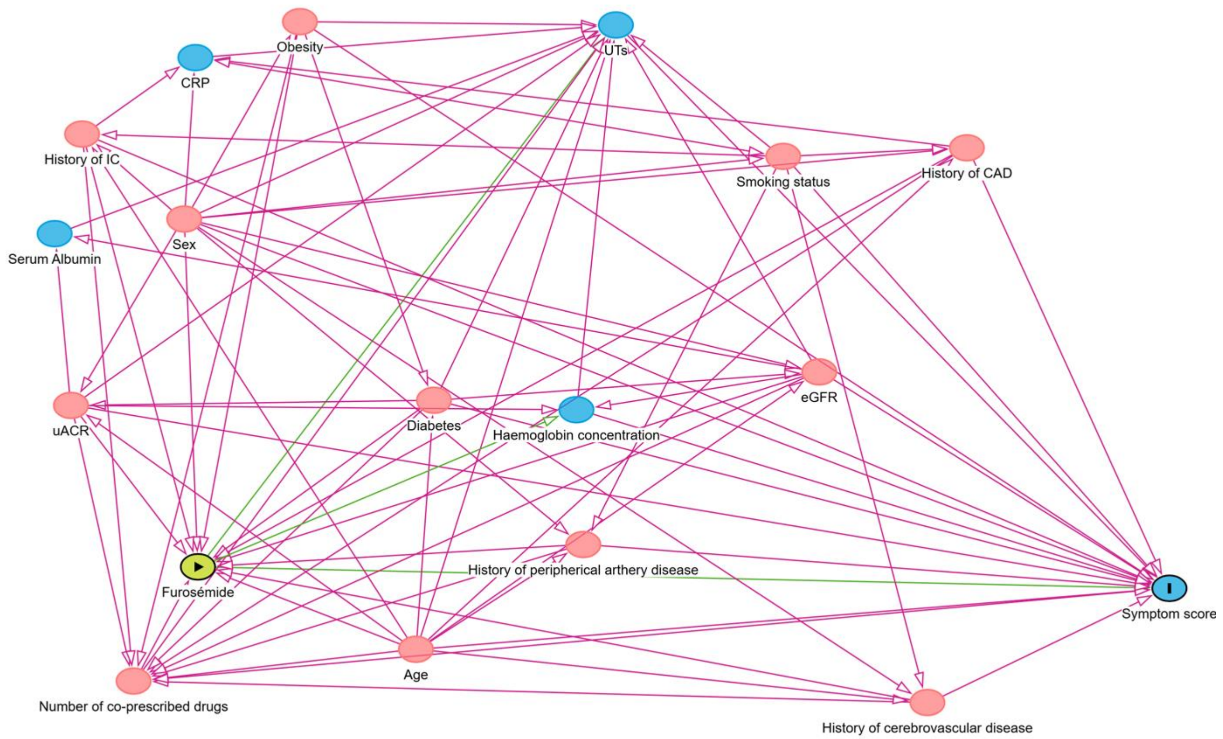

Figure S8. Directed acyclic graphs.
